# Integrated cytological and transcriptomic analyses reveal the molecular basis of spur variation in *Impatiens uliginosa*

**DOI:** 10.1186/s12870-026-09112-6

**Published:** 2026-06-03

**Authors:** Yang Liu, Xiaoli Zhang, Bo Hui, Luqiu Zhao, Hongyao Liao, Zhijia Gu, Meijuan Huang, Haiquan Huang

**Affiliations:** 1 College of Landscape Architecture and Horticulture Sciences, Yunnan Key Laboratory of Landscape Plant Resource Cultivation and Application, Yunnan Province Engineering Research Center for Functional Flower Resources and Industrialization, Research and Development Center of Landscape Plants and Horticulture Flowers, Southwest Forestry University, Kunming, 650224 China; 2 Key Laboratory for Plant Biodiversity and Biogeography of East Asia, Kunming Institute of Botany, Chinese Academy of Sciences, Kunming, 650201 China

**Keywords:** *Impatiens uliginosa*, Variant spur, Spur development, Spur morphology, Transcriptome

## Abstract

**Background:**

The spur, a key morphological structure in *Impatiens uliginosa*, plays important functional roles in attracting specific pollinators, enhancing pollination efficiency, and facilitating interspecific reproductive isolation. While its ecological and evolutionary significance has been extensively studied, the cytological and molecular mechanisms underlying spur morphogenesis, particularly in non-model plants, remain poorly understood. In this study, we used wild-type (WT) plants bearing a single spur, two-spur mutant (2SM) and three-spur mutant (3SM) of *I. uliginosa*. By integrating morphometric analysis, cytological examination, and transcriptome sequencing, we identified candidate genes and hormonal regulatory networks associated with spur variation at the molecular level for the first time. This study provides new insights into the molecular basis of spur formation in *I. uliginosa* and the genus *Impatiens* more broadly.

**Results:**

The developmental dynamics of spurs were analyzed both in WT and mutant *I. uliginosa*, indicating that spur growth follows a typical sigmoidal curve, with lateral spurs being significantly shorter than the main spurs. The cellular development mechanisms were similar between these two spur types: initial spur formation was predominantly driven by cell division, whereas subsequent elongation primarily depended on cell expansion, with the formation of internal cellular protrusions regulated by anisotropic cell growth. Transcriptome sequencing of spurs at the early stage yielded 32.69 Gb of high-quality data, from which 42,721 unigenes were assembled. Functional annotation against the NR, Swiss-Prot, Pfam, COG, GO, and KEGG databases resulted in the annotation of 24,031 genes. Differential expression analysis identified 8,592 differentially expressed genes (DEGs), which were enriched in 371 GO terms and 111 KEGG pathways. Notably, the “plant hormone signal transduction” pathway showed the highest enrichment in the mutant spurs. A total of 955 transcription factors (TFs) belonging to 34 families, including MYB, AP2/ERF, and TCP, were identified. Through qRT-PCR validation, ten candidate genes potentially involved in spur variation were identified, including *LTP, GRP5*, *PDF1*, and *GID2*.

**Conclusions:**

Our study elucidates the morphological and cellular developmental mechanisms of the spurs in WT and mutant *I. uliginosa*, and identifies a series of candidate genes associated with spur variation, including cell cycle, cell division, cell elongation, and plant hormones. The findings provide valuable data and resources for further unraveling the molecular mechanisms underlying spur variation in *Impatiens* species.

**Supplementary Information:**

The online version contains supplementary material available at https://doi.org/10.1186/s12870-026-09112-6.

## Introduction

The spur—a tubular, saccate, or similarly shaped extension formed by the backward or lateral elongation of petals or sepals—is an evolutionary novelty that serves as an important taxonomic character in plants. It is widespread across multiple families, such as Papaveraceae (e.g., *Corydalis*, *Fumaria*), Ranunculaceae (e.g., *Consolida*, *Delphinium*, *Aquilegia*, *Urophysa*), Balsaminaceae (*Impatiens*), Violaceae (e.g., *Afrohybanthus*, *Viola*), and Orchidaceae (e.g., *Calanthe*, *Dendrobium*, *Neottianthe*, *Orchis*) [1, 2]. Typically involved in nectar secretion or storage, or merely displaying a nectariferous appearance, the spur reduces nectar evaporation, enhances pollinator specificity and efficiency, and plays a key role in pollination ecology, thereby constituting a central component of the pollination syndrome hypothesis [3].

Studies show that spur morphology results from the coordinated processes of cell division and elongation. In *Centranthus ruber*, epidermal cell division contributes to roughly 30% of the final spur length during early development, after which anisotropic elongation drives subsequent growth [4, 5]. *Aquilegia* follows a comparable two-phase “division-then-elongation” pattern: localized cell division forms the nectary cup and stops once the spur attains a length of about 1 mm; thereafter, nearly all further elongation results from highly oriented cell extension, with the duration of this elongation phase shaping interspecific differences [6–8]. By contrast, spur length differences between sister species of *Linaria* stem primarily from variations in cell division rates and initial cell numbers, with no significant differences observed in mature cell anisotropy—suggesting that distinct developmental mechanisms can evolve across lineages [9]. Investigating spur cytology in *I. uliginosa*, Li et al. (2024) reported that early development is dominated by cell division, which initiates spur outgrowth from the labellum, whereas later development is marked by cell elongation that ultimately determines final spur length [10].

The coordinated development of the floral spur is governed by hormonal signaling and core TFs. Transcriptomic analysis of *I. uliginosa* indicates that pathways involved in plant hormone signal transduction are consistently enriched throughout spur development, where genes associated with the cell cycle, division, and elongation modulate spur morphology through auxin, brassinosteroid, and other hormonal pathways [11]. In Aquilegia, auxin-related genes (*ARF8*, *SAUR*, *YUCCA6*) and brassinosteroid signaling genes (*TCP4*, *CYP71A*) exhibit early differential expression and have been co-opted for novel roles: *AqTCP4* suppresses distal cell division, facilitating localized invagination around the nectary to initiate spur formation, while cooperative regulatory modules containing *ARF* and *STY* further refine hormone-directed cell proliferation and expansion [8, 12]. Concurrently, TFs including *KNOX*, *POP*, and *bHLH* constitute an evolutionarily conserved yet flexible regulatory network. In Antirrhinum majus, for instance, a transposon insertion in *HIRZINA/INVAGINATA*—a KNOX-class gene—can induce spur-like outgrowths on typically spurless petals. Orthologs of this gene are highly expressed in *Linaria vulgaris* and *Dactylorhiza fuchsii*, and their ectopic expression in tobacco similarly produces saccate structures [13–15]. In contrast, spur development in Papaveraceae proceeds independently of *STM*-like *KNOX* genes, suggesting alternative genetic mechanisms [16]. In *Aquilegia*, the C₂H₂ zinc-finger TF POPOVICH functions as a master regulator of spur presence or absence, with downstream effectors such as *ARF6/8* controlling variation in spur length [17]. Meanwhile, in *Tropaeolum majus*, lineage-specific duplications of *TCP3/4L* and *STM* genes, combined with early differential expression of bHLH027/046/082/083/092, drive cell division and spur morphogenesis [18]. Collectively, these findings demonstrate that hormone signaling and TF networks interact in lineage-specific manners to establish the molecular foundation of spur development.

Although floral spurs have been studied extensively, prior work has concentrated largely on developmental and pollination biology, leaving variation in spur morphology relatively unexplored. During field surveys of *Impatiens* populations, we observed mutants of *I. uliginosa* that produce flowers with two or three spurs. This study used WT, 2SM and 3SM of *I. uliginosa* as experimental materials. We analyzed growth dynamics, internal spur cellular structure, transcriptomic profiles, and validated candidate genes using qRT-PCR to investigate the mechanisms underlying spur number variation. Our findings are expected to provide foundational insights and a theoretical framework for floral trait modification and cultivar development in *Impatiens*.

## Results

### Spur development of *I. uliginosa*

**Fig. 1:**
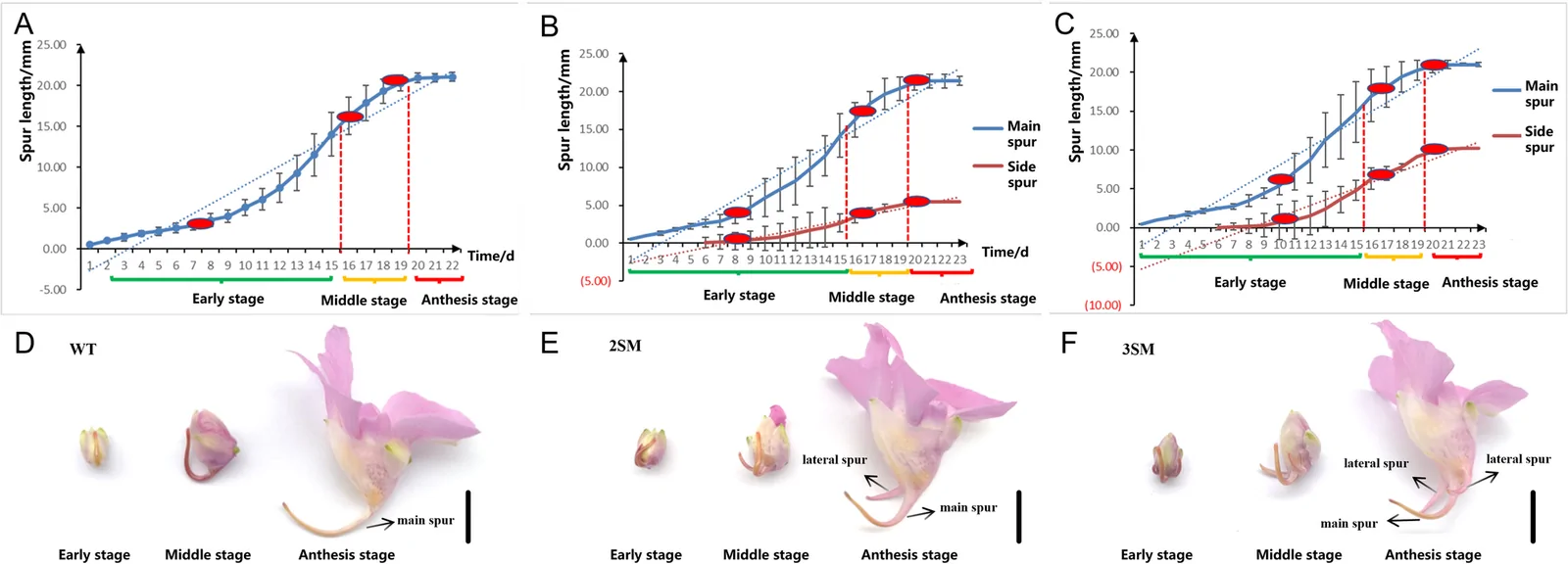
Spur development of *I. uliginosa*. **A** Average growth curve of WT. **B** Average growth curves of the main spurs and lateral spurs in 2SM. **C** Average growth curves of the main spurs and lateral spurs in 3SM*.* The red dashed line indicates the boundary between the three developmental stages. **D**, **E** and **F** Spurs in the early stage, showing a straight appearance (left); Spurs in the middle stage, producing an inward curve (middle); Spurs at anthesis stage (right). Scale bar = 1 cm

Based on morphological traits and growth curves, the development of both WT and mutant floral spurs can be categorized into three distinct stages: early stage, middle stage, and anthesis stage (Fig. 1A–C). The primary spurs of WT *I. uliginosa*, along with the main spurs of 2SM and 3SM, displayed highly similar growth dynamics, completing development within 22–23 days and following a characteristic sigmoidal (“S”-shaped) growth curve. Rapid elongation commenced during the early stage, slowed by the middle stage, and plateaued after the anthesis stage, with final spur lengths ranging from 18 to 23 mm. Lateral spurs in 2SM and 3SM initiated growth 6–8 days after the primary spurs. While their growth patterns were similar to those of the primary spurs, lateral spurs were significantly shorter. The lateral spurs of 2SM reached approximately 5 mm, whereas those of 3SM attained about 10 mm (Fig. 1D–F).

### The development of spurs in *I. uliginosa* involves both cell division and anisotropic cell elongation

**Fig. 2:**
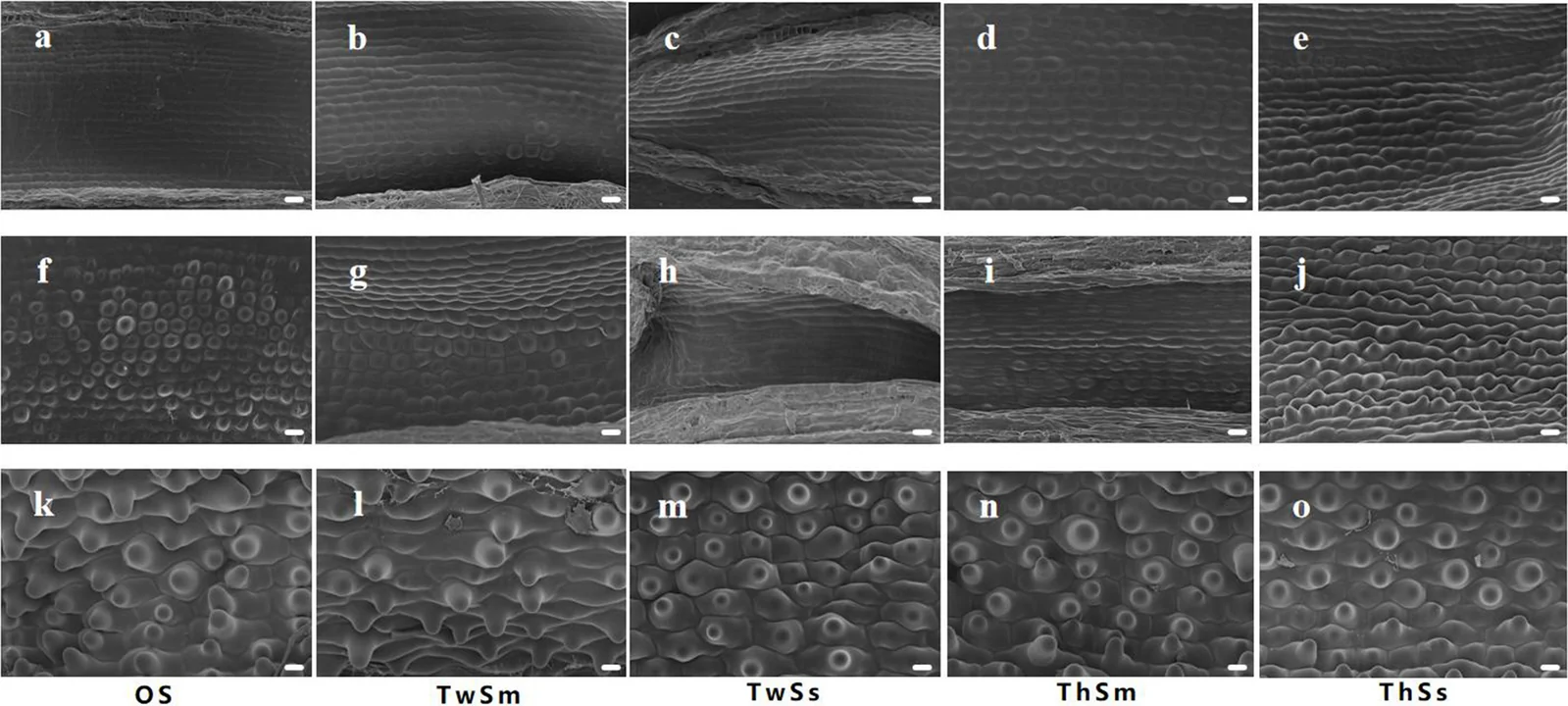
Internal structure of the nectar spurs in WT and mutant *I. uliginosa* at different developmental stages. Scale bar = 20 μm. a-e represent early stage, f-j represent middle stage, k–o represent anthesis stage. OS, WT; TwSm, main spurs of 2SM; TwSs, lateral spurs of 2SM; ThSm, main spurs of 3SM; ThSs, lateral spurs of 3SM

To elucidate the mechanisms driving spur variation in WT and mutant *I. uliginosa*, we analyzed the internal cellular architecture of spurs across three critical developmental phases: early stage, middle stage, and anthesis stage. Across these stages, the cellular morphology of all spur types transitioned progressively from a smooth to a papilla-like form (Fig. 2). During the early stage, internal cells of WT spurs were flattened and texturally uniform. Similarly, the main and lateral spurs of both 2SM and 3SM showed relatively smooth internal cells, with no marked morphological differences detectable among the spur types at this phase (Fig. 2: OS, TwSm, TwSs, ThSm, ThSs). Upon progression to the middle stage, WT spur cells started to bulge. In 2SM, main and lateral spurs presented distinct layered and wrinkled textures. In contrast, the main and lateral spurs of 3SM developed irregularly spaced protrusions and a generally looser cellular organization (Fig. 2: MOS, MTwSm, MTwSs, MThSm, MThSs). By the anthesis stage, protrusions on WT spurs had become more rounded and densely arranged. The structural complexity increased further in both mutant types, characterized by an interwoven network of wrinkles and protrusions (Fig. 2: AOS, ATwSm, ATwSs, AThSm, AThSs).

**Fig. 3:**
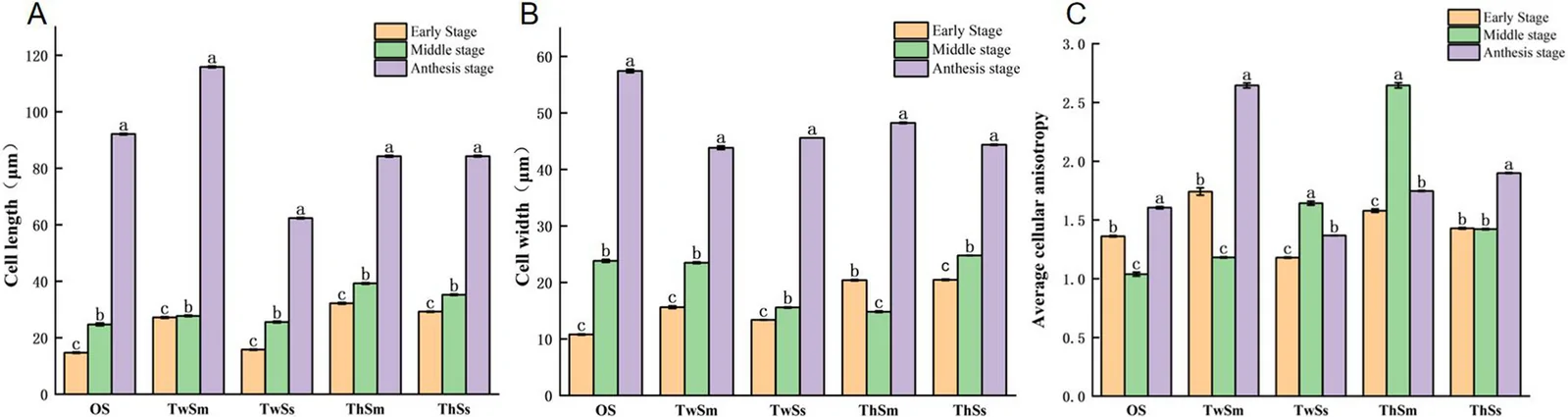
Statistical analysis of cell length, cell width and average cell anisotropy of WT and mutant *I. uliginosa.* OS, WT; TwSm, main spurs of 2SM; TwSs, lateral spurs of 2SM; ThSm, main spurs of 3SM; ThSs, lateral spurs of 3SM

Based on scanning electron microscopy (SEM) images, we conducted statistical analyses of cell length, cell width, and mean anisotropy within the floral spurs (Fig. 3). The results showed that from the early stage to the anthesis stage, both cell length and cell width increased significantly in WT and mutant spurs (*P* < 0.05) (Fig. 3A, B). However, at the same developmental stage, significant differences were observed in cell length, cell width, and anisotropy among different spur types. Specifically, at the early stage, significant differences were detected in spur length, spur width, and anisotropy among different types (*P* < 0.05); at the middle stage, significant differences were found in spur length and anisotropy among different types (*P* < 0.05), whereas no significant difference in cell width was observed between OS and TwSm; at the anthesis stage, significant differences in spur length were detected among OS, TwSm, and TwSs (*P* < 0.05), and significant differences were also observed in cell width and cell anisotropy among different types (*P* < 0.05).

**Fig. 4:**
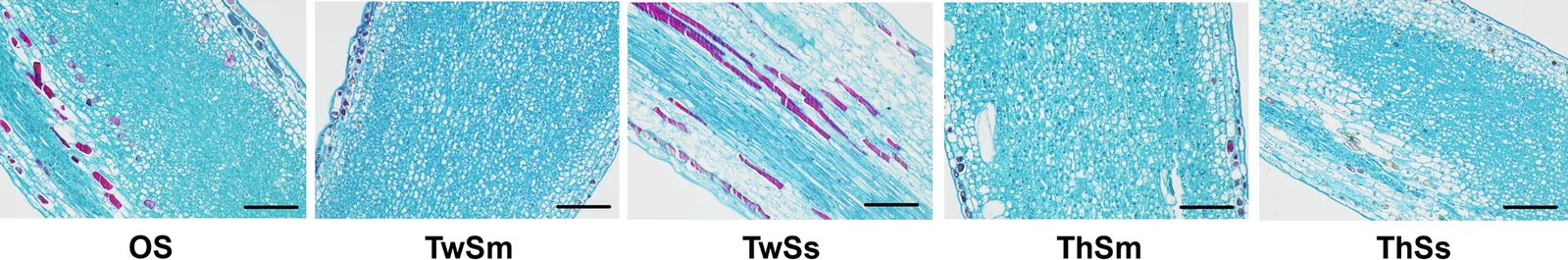
Epidermis of the spurs in the WT and mutant *I. uliginosa* at the anthesis stage. OS, WT; TwSm, main spurs of 2SM; TwSs, lateral spurs of 2SM; ThSm, main spurs of 3SM; ThSs, lateral spurs of 3SM. Scale bar = 100 μm

As the floral spurs of both WT and mutant *I. uliginosa* were fully developed at the anthesis stage, we performed histological examinations and quantified their internal cell number and cell area (Fig. 4, Table 1). All five spur types across WT and mutant plants contained the same cell types, with densely arranged nucleated cells and vascular bundle tissues potentially interspersed among the epidermal cells (Fig. 4). The main spurs of both WT and mutants possessed a significantly greater number of cells than mutant lateral spurs, suggesting that cell number may be a primary factor underlying the length disparity between main and lateral spurs. In contrast, the internal cell area showed no significant differences among the different spur types (Table 1).

**Table 1:** Statistical data on spur cell morphology in WT and mutant *I. uliginosa*

Sample ID	Number of cells	Cell area/μm^2^
OS	446 a	13,031.4 a
TwSm	463 a	12,507.25 a
TwSs	254 b	11,722.33 a
ThSm	424 a	11,719.86 a
ThSs	254 b	10,498.86 a

Based on wax section images of the middle outer epidermis of the nectar spurs from WT and mutant *I. uliginosa* at the anthesis stage, the sample area, cell number, and mean cell anisotropy data were measured at 400 × magnification

By integrating scanning electron microscopy with histological analysis, we conclude that the cellular developmental mechanisms of floral spurs in WT and mutant *I. uliginosa* are fundamentally similar: early spur formation depends primarily on cell division, whereas later elongation is driven mainly by cell expansion. The formation of internal papillary cell structures and the morphological variation among spurs appear to be regulated principally by cellular anisotropy.

### Transcriptome RNA sequencing and De Novo assembly

Transcriptomic analysis was performed on floral spurs at the early stage, specifically focusing on five distinct groups: WT spurs (OS), main spurs of 2SM (TwSm), lateral spurs of 2SM (TwSs), main spurs of 3SM (ThSm), and lateral spurs of 3SM (ThSs). A total of 230,870,736 raw reads were obtained, amounting to approximately 32.69 Gb of sequencing data. After quality control, each sample yielded approximately 6.11 Gb of high-quality clean data, consisting of roughly 40 million valid reads. The Q30 scores of the reads ranged from 94.36% to 94.50%, and the GC content exceeded 43.49% in all cases. The sequencing alignment coverage for all five samples was above 87.13% (Table 2).

**Table 2:** Summary of sequencing data of *I. uliginosa* transcriptome

Attributes	OS	TwSm	TwSs	ThSm	ThSs
Raw reads	47,634,206	43,413,662	46,311,164	46,773,286	46,738,418
Clean reads	45,455,420	41,650,530	44,664,562	45,210,178	45,028,386
Clean bases	6,685,598,669	6,109,376,886	6,594,787,929	6,669,815,808	6,635,220,780
Q30(%)	94.36	94.48	94.46	94.50	94.42
GC content(%)	43.97	43.69	43.86	43.49	43.67
Total mapped	19,931,464	18,173,957	19,485,808	19,695,150	19,731,553
	(87.70%)	(87.27%)	(87.25%)	(87.13%)	(87.64%)

**Fig. 5:**
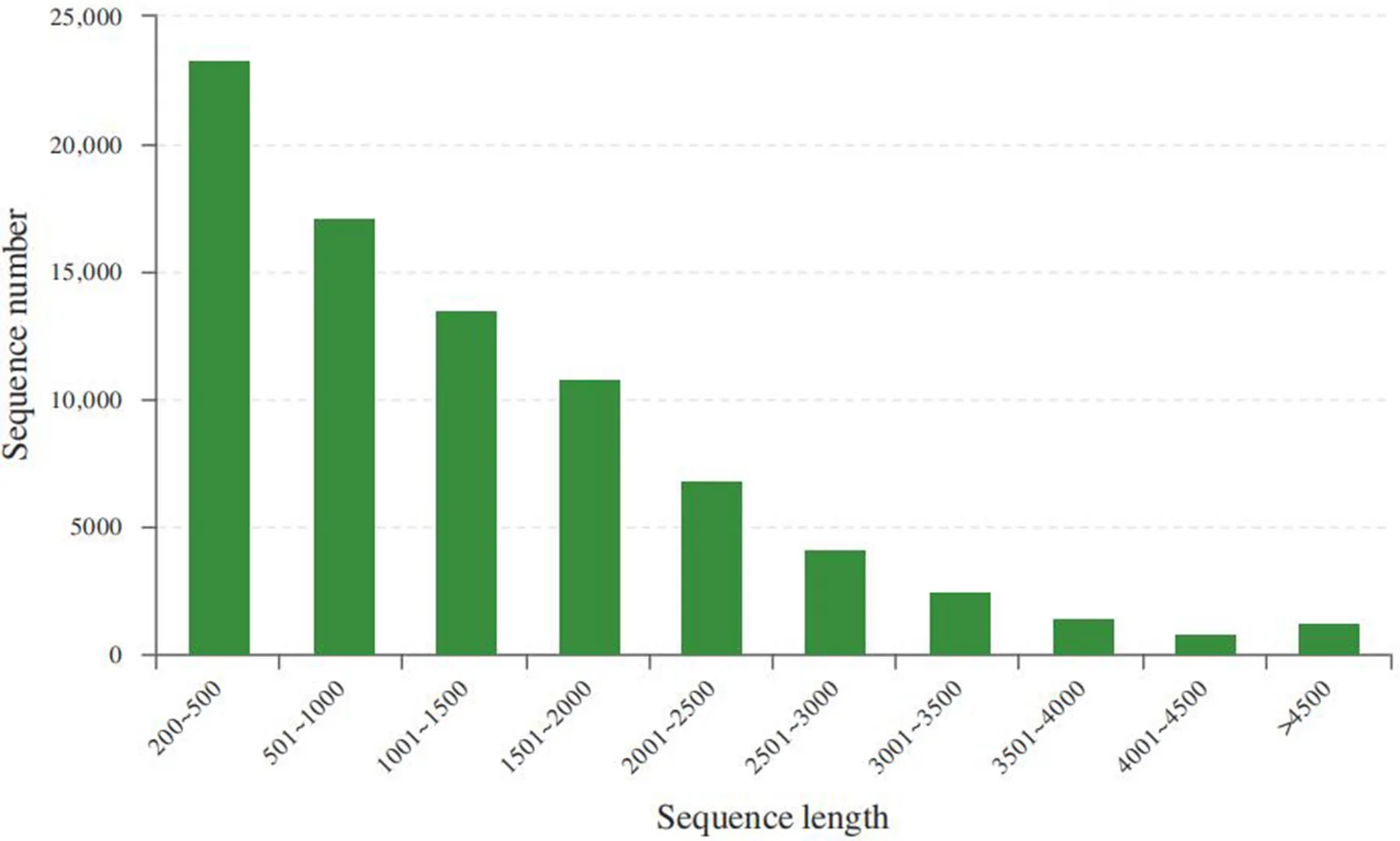
Length distribution of unigenes

Following de novo assembly based on high-quality sequencing data, 81,512 transcripts with a combined length of 104.9 Mb were obtained after optimization and filtering. The transcripts ranged in length from 201 bp to 13,223 bp, with a mean length of 1,287.05 bp and an N50 of 1,910 bp. Subsequently, 42,721 unigenes (total length 46.9 Mb) were generated. Their maximum and minimum lengths were consistent with those of the transcripts, with a mean length of 1,097.13 bp and an identical N50 of 1,910 bp (Table 3). Unigene length distribution analysis indicated that 83.59% were 200–2,000 bp long, 15% were 2,000–4,000 bp, and an additional 998 unigenes (2.34%) exceeded 4,000 bp (Fig. 5).

**Table 3:** Statistics of transcriptome assembly

Attributes	unigenes	transcripts
Total number	42,721	81,512
Total base	46,870,323	104,909,795
Largest length (bp)	13,223	13,223
Smallest length (bp)	201	201
Average length (bp)	1,097.13	1,287.05
N50 length (bp)	1,910	1,910

### Functional annotation

Of the 42,721 assembled unigenes, 24,031 (56.25%) were successfully annotated based on alignments against six major databases (Table 4, Table S1). Among these annotated unigenes, 23,818 (55.75% of the total) matched entries in the NR database; of these, 47.93% (11,417 unigenes) exhibited sequence similarity greater than 80%, and 18,828 (79.05%) showed high homology (E-value < 1 × 10⁻^3^⁰). The five species with sequences most similar to those of *I. uliginosa* were *Camellia sinensis* (5,058 matches; 21.24%), *Actinidia chinensis* (2,780; 11.67%), *Nyssa sinensis* (2,575; 10.81%), *Actinidia rufa* (968; 4.06%), and *Vitis vinifera* (682; 2.86%) (Fig. S1). Furthermore, 18,989 (44.32%), 18,326 (42.90%), 21,780 (50.98%), 20,568 (48.14%), and 10,405 (24.36%) unigenes were aligned to the Swiss-Prot, Pfam, COG, GO, and KEGG databases, respectively.

**Table 4:** Functional annotation of *I. uliginosa* unigenes

Database	Number of unigenes	Percentage
NR	23,818	55.75%
Swiss-Prot	18,935	44.32%
Pfam	18,326	42.90%
COG	21,780	50.98%
GO	20,568	48.14%
KEGG	10,405	24.36%
Total	24,031	56.25%

**Fig. 6:**
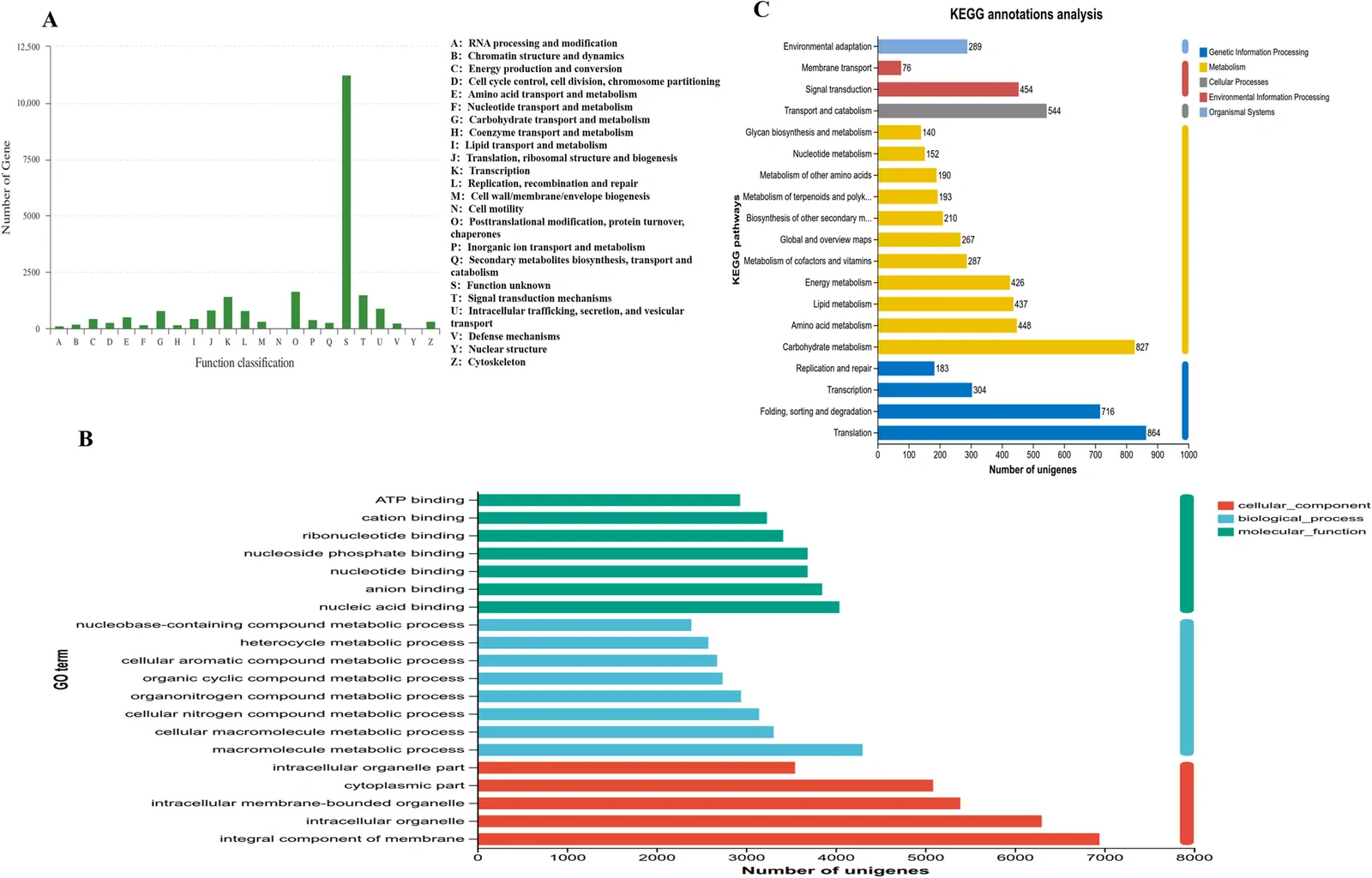
**A** COG classification. **B** Main GO categories. **C** KEGG metabolic pathways of unigenes in *I. uliginosa*

A total of 21,780 unigenes were assigned to 23 COG categories, with 11,249 remained unannotated. Among the annotated unigenes, the largest COG category was "Posttranslational modification, protein turnover, chaperones," followed by "Signal transduction mechanisms" and "Transcription" (Fig. 6A). In total, 20,568 unigenes were annotated against the GO database and classified into three main GO categories: Biological Process (BP), Cellular Component (CC), and Molecular Function (MF). Within the BP class, "macromolecule metabolic process" and "cellular macromolecule metabolic process" contained the highest number of genes, with 4,303 and 3,310 assignments, respectively. In the CC class, 6,947 unigenes were assigned to "integral component of membrane." At the MF level, the most prominent subcategories were "nucleic acid binding" and "anion binding" (Fig. 6B), encompassing 4,044 and 3,851 unigenes, respectively. Additionally, 10,405 unigenes were mapped to 19 pathways across five major KEGG categories. The most representative pathways included "Carbohydrate metabolism," "Translation," "Folding, sorting and degradation," and "Transport and catabolism" (Fig. 6C).

### Differential gene expression

**Fig. 7:**
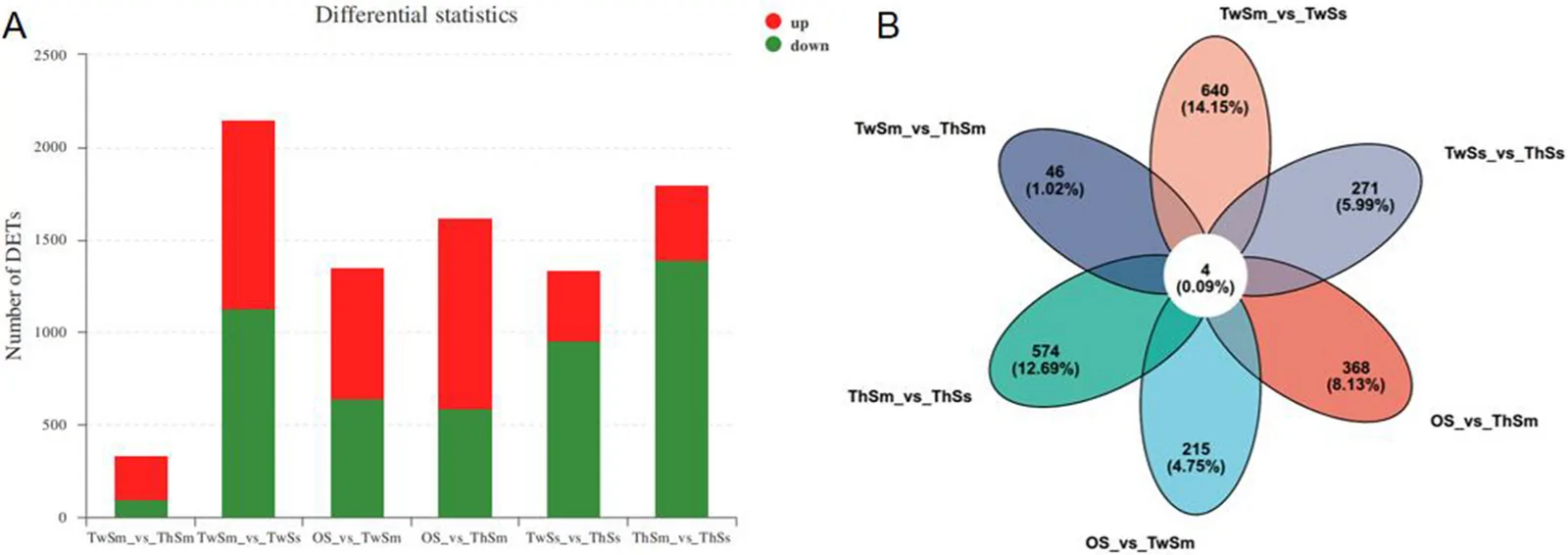
**A** Differentially expressed genes in different developmental stages and tissues. **B** Venn diagram of DEGs

To identify potential regulators of floral spur variation in *I. uliginosa*, we analyzed DEGs among different spur types at the early stage. In total, 8,592 DEGs were detected across the six pairwise comparison groups (Fig. 7A). Comparisons of the OS with TwSm and ThSm revealed 1,355 and 1,618 DEGs, respectively. Among these, 713 and 1,030 were upregulated, while 642 and 588 were downregulated. A total of 2,150 DEGs were identified between TwSm and TwSs, consisting of 1,022 upregulated and 1,128 downregulated genes. Similarly, 1,798 DEGs were found between ThSm and ThSs, with 410 upregulated and 1,388 downregulated genes. Only four DEGs were co-expressed across all these six groups (Fig. 7B).

### Enrichment analysis of DEGs

**Fig. 8:**
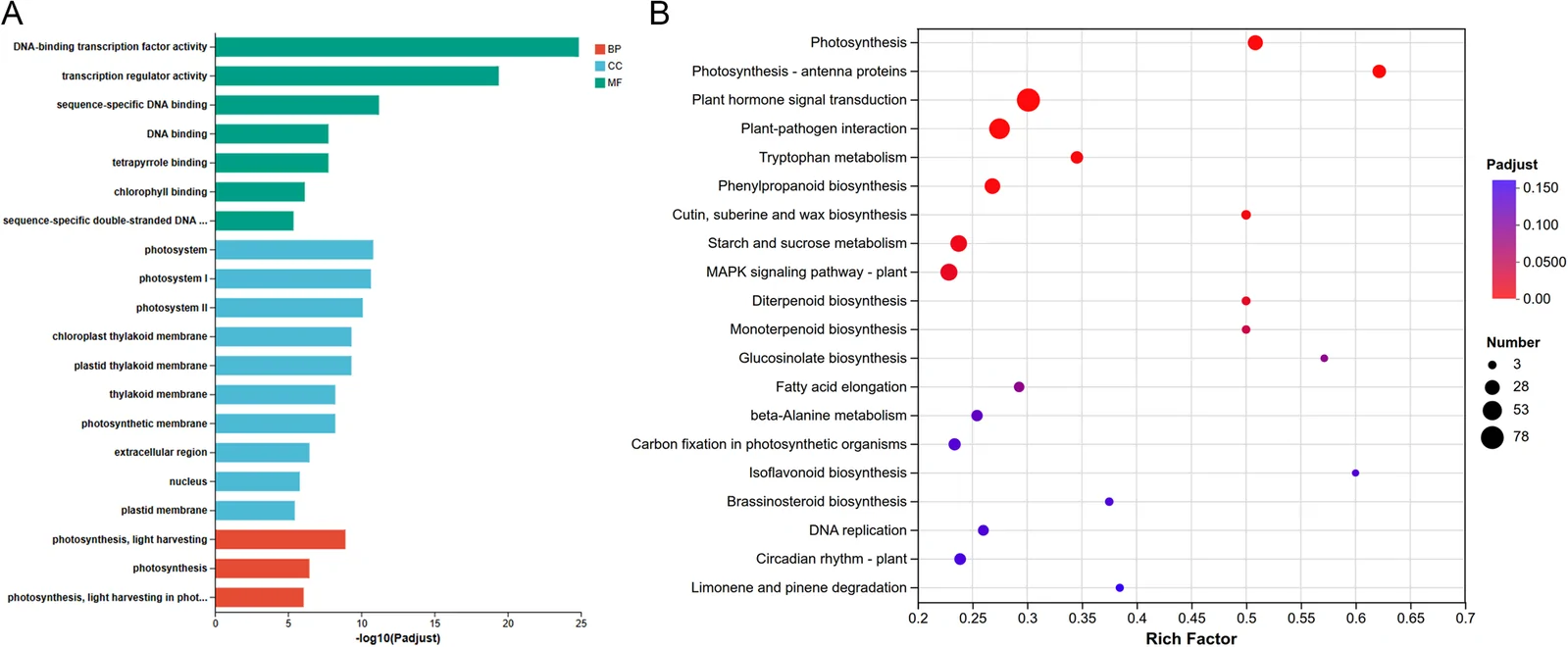
**A** Top 20 enriched GO terms of DEGs. **B** Top 20 enriched KEGG pathways of DEGs

To explore the potential functions of DEGs in floral spur variation of *I. uliginosa*, we conducted functional enrichment analysis. All 8,592 DEGs mapped to 371 Gene Ontology (GO) terms, with 30 terms significantly enriched (*P* < 0.05; Table S2). The most significantly enriched GO terms were "DNA-binding transcription factor activity," "photosystem," and "photosynthesis, light harvesting" (Fig. 8A). The enrichment profiles of DEGs varied notably among different spur types. In comparisons of main spurs—specifically, OS vs. TwSm and OS vs. ThSm—"DNA-binding transcription factor activity" was prominently enriched. Conversely, the comparison between TwSm vs. ThSm was primarily enriched in "cytochrome-c oxidase activity" and "oxidoreductase activity, acting on a heme group of donors." Among lateral spurs, the comparison between TwSs vs. ThSs was predominantly enriched for "photosystem II." Within 2SM, TwSm vs. TwSs showed enrichment of "DNA-binding transcription factor activity," while the corresponding comparison in 3SM (ThSm vs. ThSs) was mainly enriched for "plastid thylakoid membrane" and "chloroplast thylakoid membrane." Remarkably, "DNA-binding transcription factor activity" was consistently enriched across all pairwise comparisons (OS vs. TwSm, OS vs. ThSm, TwSm vs. ThSm, TwSm vs. TwSs, ThSm vs. ThSs, and TwSs vs. ThSs), implying its crucial role in spur differentiation (Fig. S2).

KEGG enrichment analysis revealed that a total of 8,592 unigenes were annotated to 111 KEGG pathways, among which 11 pathways exhibited significant enrichment (*P* < 0.05) (Fig. 8B, Table S3). Further functional enrichment analysis of DEGs derived from different spurs types indicated that "Plant-pathogen interaction," "MAPK signaling pathway—plant," "Plant hormone signal transduction," "Phenylpropanoid biosynthesis," and "Monoterpenoid biosynthesis" were consistently enriched in all spur types. Specifically, "Plant-pathogen interaction," "Plant hormone signal transduction," and "MAPK signaling pathway—plant" were significantly enriched in comparison groups related to main spurs (OS vs. TwSm, OS vs. ThSm, TwSm vs. ThSm). In contrast, "Photosynthesis—antenna proteins," "Plant hormone signal transduction," "DNA replication," and "Plant-pathogen interaction" were notably enriched in the comparison of lateral spurs comparison (TwSs vs. ThSs). Additionally, multiple metabolic and synthetic pathways, including "Starch and sucrose metabolism," "Oxidative phosphorylation," "Phenylpropanoid biosynthesis," "Monoterpenoid biosynthesis," "Tryptophan metabolism," "Brassinosteroid biosynthesis," and "Carbon fixation in photosynthetic organisms" were specifically enriched in the 2SM comparison (TwSm vs. TwSs). Meanwhile, "Photosynthesis," "Photosynthesis – antenna proteins," "Plant hormone signal transduction," "Carbon fixation in photosynthetic organisms," and "Glucosinolate biosynthesis" were uniquely enriched in 3SM comparison (ThSm vs. ThSs) (Fig. S3).

### Identification of TFs

**Fig. 9:**
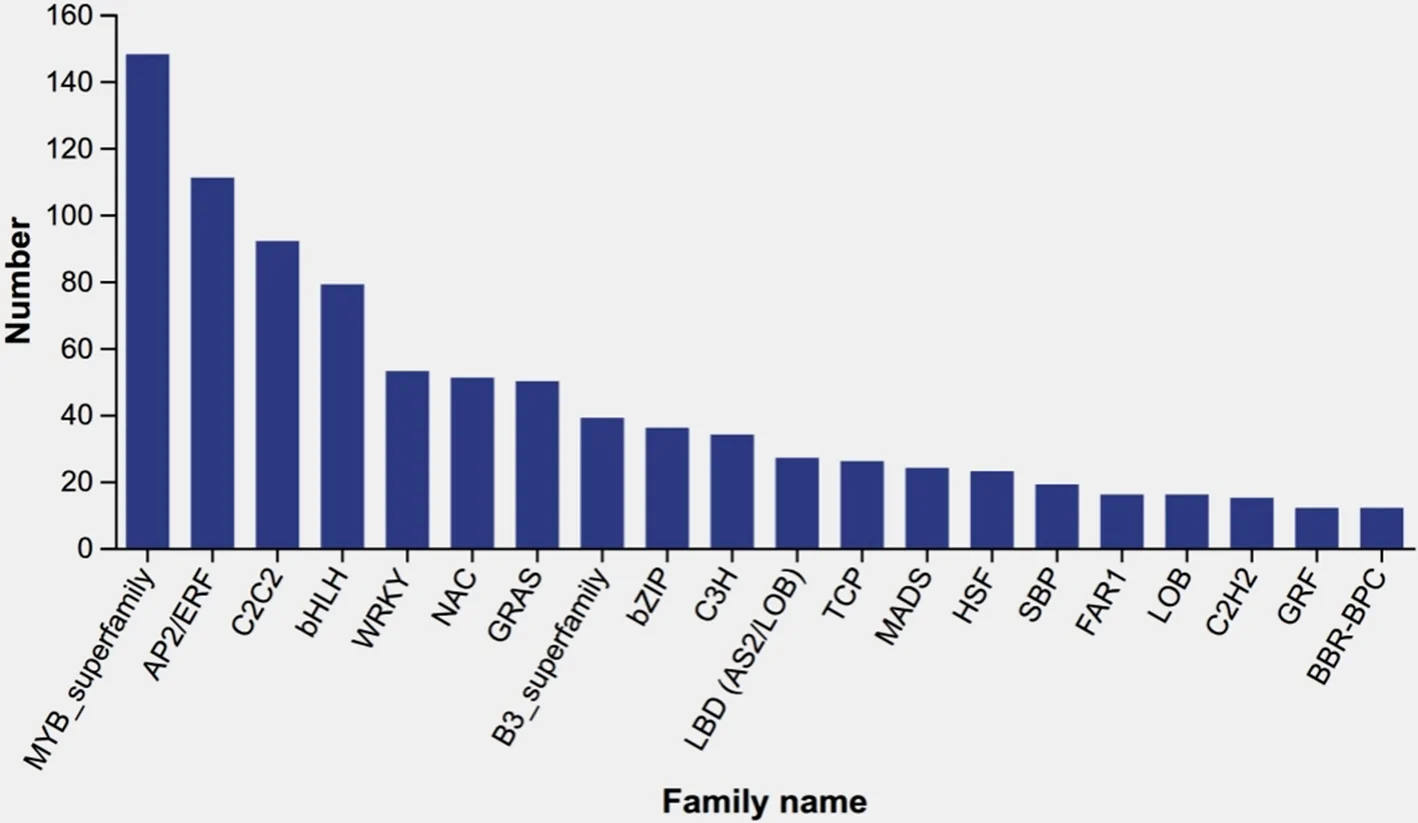
Families of TFs identified in the *I. uliginosa* transcriptome

**Fig. 10:**
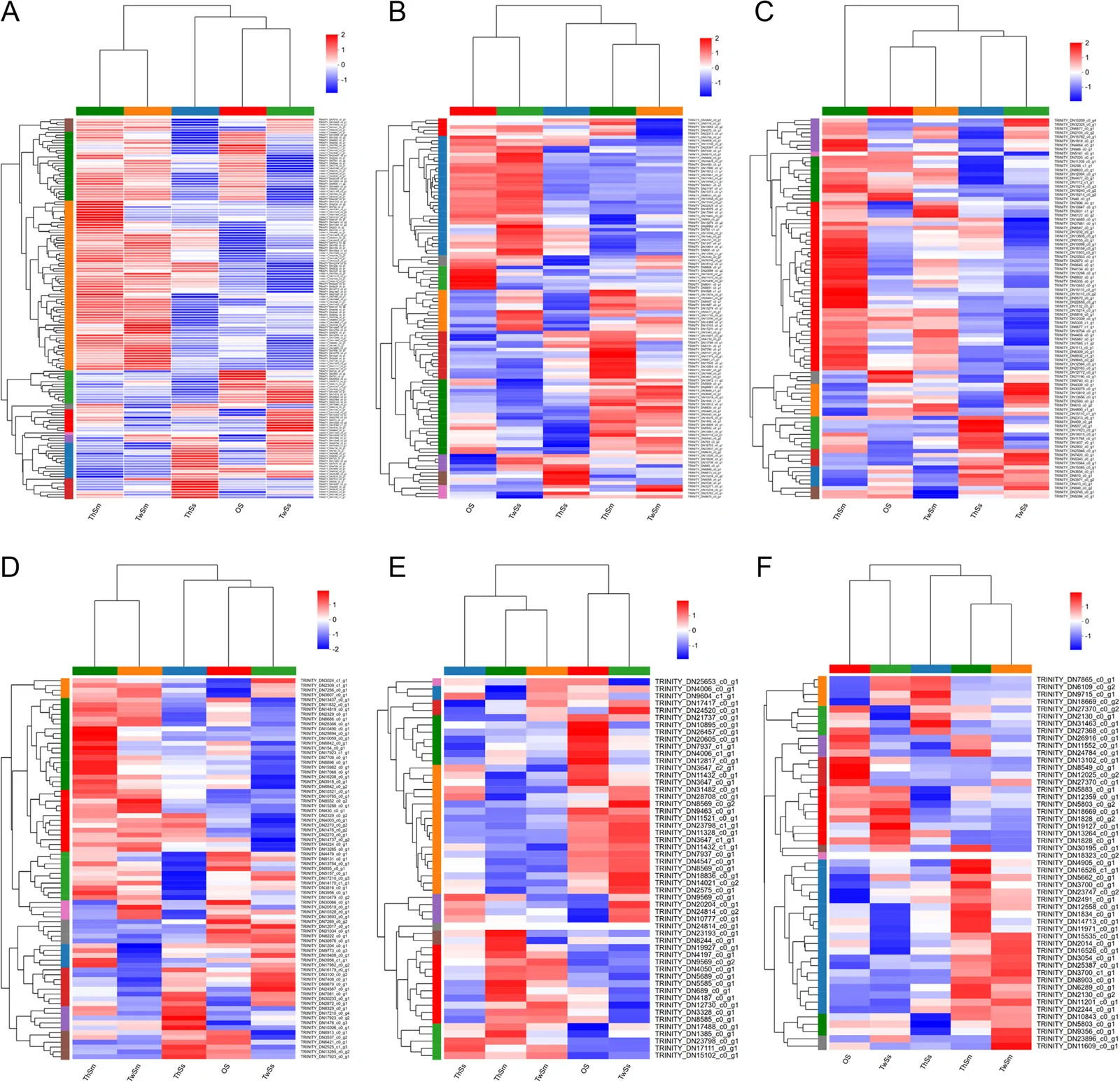
Cluster heat maps of DEGs in TF families. **A** MYB. **B** AP2/ERF. **C** C2C2. **D** bHLH. **E** WRKY. **F** NAC

TFs were predicted by analyzing domain information within the transcriptome. A total of 955 unigenes were annotated as TFs, belonging to 34 distinct families (Fig. 9). Among these, the MYB family contained the most members (148 genes), followed by AP2/ERF (111), C2C2 (92), bHLH (79), and WRKY (53). All 955 transcription factor genes were differentially expressed across different floral spurs, with expression levels varying by up to 56-fold (Table S4). Members of the MYB and bHLH families exhibited higher expression in the main spur of 2SM and 3SM (Fig. 10A, D). The AP2/ERF family showed elevated expression in the WT single spur and the main spur of mutant plants (Fig. 10B). High expression of the C2C2 family was specific to the main spur of the 3SM, while its expression was lower in the lateral spur of both the 2SM and 3SM (Fig. 10C). The WRKY family was highly expressed only in the WT spur and the lateral spur of the 2SM (Fig. 10E). Lastly, the NAC family showed relatively high expression in both the 2SM (TwSm/TwSs) and 3SM (ThSm/ThSs) mutants (Fig. 10F).

### Candidate genes involved in spur development

Based on gene functional differences, expression levels in WT and mutant *I. uliginosa*, enrichment analyses, and transcriptomic studies in other species, we preliminarily identified 10 candidate genes associated with floral spur variation in *I. uliginosa* (Table S5). These candidate genes include *GID2* (TRINITY_DN8041_c0_g1), which participates in plant hormone signal transduction; *SOB5* (TRINITY_DN4278_c0_g1), associated with cytokinin biosynthesis; and the transcription factor *NAC2* (TRINITY_DN8903_c0_g1). Expression levels of these three genes were lower in the WT than in mutant main spurs, and higher in mutant main spurs than in lateral spurs. The transcription factor *SOC1* (TRINITY_DN2881_c0_g1) and the lipid transport and metabolism gene *LTP* (TRINITY_DN11611_c0_g1) showed higher expression in the WT than in mutants. Specifically, *SOC1* expression was lower in the main spurs of 2SM than in its lateral spurs, but higher in the main spurs of 3SM than in its lateral spurs. *LTP* expression was higher in the main spurs than in the lateral spurs of 2SM, but exhibited the opposite expression trend in 3SM. The transcription factor *TCP4* (TRINITY_DN11420_c0_g1) exhibited lower expression in mutants compared to the WT — notably, *TCP4* has previously been confirmed to regulate floral spur development in *Aquilegia* and *Linaria*. Additionally, the highly expressed cell wall structural gene *GRP5* (TRINITY_DN8368_c0_g1) and the extremely highly expressed plant defensin gene *PDF1* (TRINITY_DN486_c0_g1; TPM > 2000) showed lower expression in the main spurs than in the lateral spurs of 2SM. *GRP5* expression was higher in the main spurs than in the lateral spurs of 3SM, while *PDF1* expression was lower in the main spurs than in the lateral spurs of 3SM.

### qRT-PCR validation of the candidate genes

**Fig. 11:**
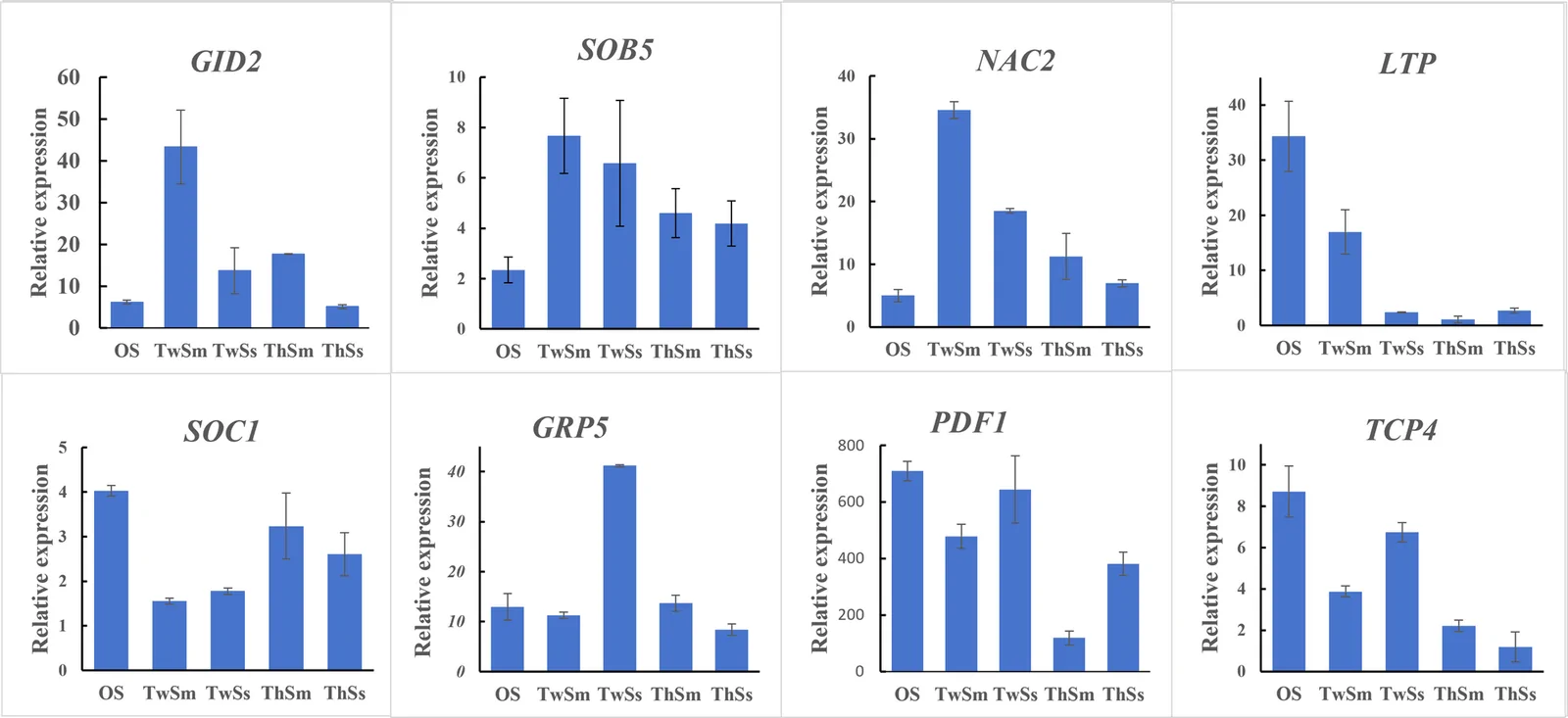
Expression analysis of eight candidate genes in 5 tissues by qRT-PCR

To validate the transcriptome data, eight candidate genes were selected for qRT-PCR analysis (Fig. 11, Table S6). qRT-PCR results showed that *GID2* expression was significantly higher in the main spurs than in lateral spurs (*P* < 0.05). *SOB5* expression was significantly lower in the WT compared with the TwSm and TwSs mutants (*P* < 0.05), while TwSm exhibited significantly higher expression levels than ThSm and ThSs (*P* < 0.05). Expression of *NAC2* was elevated across all mutant types relative to the WT, with TwSm, TwSs, and ThSm showing significantly higher levels than OS (*P* < 0.05). Conversely, the expression levels of *LTP* and *TCP4* were significantly higher in the WT than in the mutants (*P* < 0.05). *GRP5* expression was uniquely and significantly elevated in the TwSs compared to all other tissues (*P* < 0.05). While *SOC1* expression was generally higher in the WT than in the mutants, the difference was significant when compared with TwSm, TwSs, and ThSs. Finally, among the mutants, *PDF1* expression was significantly higher in lateral spurs than in the main spurs (*P* < 0.05).

## Discussion

The floral spur is a hollow tubular structure that typically functions as a nectar reservoir. It represents both an evolutionary innovation and an important taxonomic trait in plants. Numerous plant taxa exhibit distinctive spurs, including *Aquilegia* and *Consolida* (Ranunculaceae), *Linaria* (Scrophulariaceae), and certain *Cymbidium* species (Orchidaceae). Spurs enhance pollination efficiency and reproductive success, contribute to morphological diversification [19], and may influence mechanisms related to biological invasion [20, 21]. Consequently, the floral spur is considered a key innovation trait, and its development and biological function have attracted substantial research attention. Most species of the genus *Impatiens* bear floral spurs, and their wide interspecific morphological variation renders this genus an ideal model for exploring spur biology. Earlier cytological studies showed that during early development, the spur of *I. uliginosa* displays prominent cell aggregation and active division compared to other regions of the labellum [11]. Before spur differentiation, the spur primordium initiates intense cell division, which establishes the basis for subsequent morphogenesis and drives the outward extension of the spur from the labellum [10]. In the present study, we observed a significant increase in cell number in the spurs of both WT and mutant *I. uliginosa* from the early to middle developmental stage, followed by marked cell elongation from early stage to anthesis stage. This developmental pattern closely parallels that reported in *Aquilegia* and *Centranthus ruber*, where spur growth relies on sequential cell division and anisotropic elongation, with cellular elongation acting as the principal driver of spur elongation [5, 6]. Differentially expressed genes between the spur and the limb tissues potentially participate in the regulation of spur development [10, 11].

Comparative transcriptomic analysis of five floral spur types in *I. uliginosa* at the early stage systematically uncovered key molecular mechanisms underlying spur morphogenesis. We identified 8,592 DEGs, of which four were differentially expressed in every pairwise comparison, implying their potential roles as core regulators of spur development. Enrichment analysis revealed significant DEG enrichment in pathways including “Plant-pathogen interaction,” “MAPK signaling pathway – plant,” “Plant hormone signal transduction,” “Phenylpropanoid biosynthesis,” and “Monoterpenoid biosynthesis” in comparisons between WT and mutant spurs. These results indicate that early spur development involves active cell division, growth, and specialized secondary metabolism, which is consistent with previous studies on *I. uliginosa*, *Aquilegia*, and *Linaria* [8, 9, 11, 22]. In comparisons between the WT spur and mutant main spurs, pathways such as “Plant-pathogen interaction,” “Plant hormone signal transduction,” and “MAPK signaling pathway – plant” remained consistently enriched, suggesting that main spurs sustain vigorous elongation alongside fundamental immune and signaling networks. By contrast, DEGs derived from lateral spurs were prominently enriched in “Photosynthesis – antenna proteins,” “Plant hormone signal transduction,” “DNA replication,” and “Plant-pathogen interaction,” indicating that lateral spurs exhibit active cell division, rapid elongation, and enhanced photosynthetic capacity. Notably, “Plant hormone signal transduction” was significantly enriched across all spur-type comparisons. This finding underscores the plant hormone signaling network as a central regulatory hub that coordinates morphological variation in *I. uliginosa* spurs, likely by integrating developmental and defense signals to ultimately shape distinct spur morphologies.

TFs act as central hubs within gene regulatory networks and are pivotal in shaping complex plant traits. In I. uliginosa, we identified 955 differentially expressed TFs representing 34 families. All TFs were differentially expressed across the different spur types, suggesting their potential roles in regulating spur development. The AP2/ERF family exhibited elevated expression in the WT single spur and the main spurs of mutants, suggesting its involvement in early spur development and cell cycle regulation, and implying a critical role in spur differentiation. The AP2/ERF family functions as a global regulator of plant development and stress responses, integrating multiple signals via its “identity determination–cell differentiation–senescence regulation” network [23, 24]. The MYB and bHLH families were highly expressed specifically in the main spurs of 2SM and 3SM mutants, indicating divergent developmental mechanisms between WT spurs and mutant main spurs. The MYB family, with its dual “activation–repression” regulatory mode, acts as a hub for spatiotemporal precision in organ development [25], whereas bHLH factors are critical for processes such as organogenesis and pigment deposition [26]. The WRKY family was highly expressed specifically in the lateral spurs of the WT and 2SM mutant, indicating divergent growth and developmental mechanisms between lateral and main spurs, and suggesting that this family may represent one of the key factors contributing to spur variation and the formation of multiple spurs in I. uliginosa. The WRKY family is among the largest plant-specific transcription factor families. In addition to directly regulating flowering and pigment synthesis genes to determine floral organ morphology and coloration, they function as central hubs within signaling networks to integrate environmental cues with endogenous developmental programs, thereby precisely regulating the transition from vegetative to reproductive growth [27, 28]. The NAC family was highly expressed in all four spurs of the 2SM (TwSm/TwSs) and 3SM (ThSm/ThSs) mutants, suggesting its involvement in the morphogenesis of mutant spurs. NAC transcription factors modulate plant architecture via the 'cell wall–transport–senescence' module and govern flowering time and morphological development through a four-tier regulatory cascade encompassing floral transition, organ boundary establishment, anther dehiscence, and perianth display, thereby functioning as key molecular switches for plant growth, development, and reproductive success [29, 30]. The C2C2 family exhibited both significant differential expression and high expression levels across different spur types in both WT and mutant plants, consistent with previous studies in spurred plants.

To identify potential regulators of floral spur variation in *I. uliginosa*, 10 candidate genes were screened, eight of which were validated via qRT-PCR. This validation confirmed the reliability of the transcriptome data. All eight genes exhibited significantly differential expression across distinct spur types in both WT and mutant plants.The GID2 gene, which participates in the gibberellin (GA) signaling pathway, functions in plant growth and development by coordinating with other *GID* family proteins to regulate cell division and elongation [31]. In this study, *GID2* expression was significantly higher in the main spurs compared with lateral spurs, suggesting that it may contribute to spur elongation and could partially explain why main spurs are longer than lateral spurs. Both *GRP5* and *PDF1* are implicated in growth, defense, and stress responses. *GRP5* encodes a glycine-rich protein likely involved in stress response and cell-wall maintenance [32]. *PDF1*, a membrane component, shows preferential expression during Gossypium hirsutum fiber initiation and early elongation, where *GbPDF1* interacts with *GhMYB25-like* to modulate H–O homeostasis, ethylene signaling, and pectin biosynthesis [33]. Here, *GRP5* expression was significantly upregulated in TwSs, whereas *PDF1* showed lower expression in main spurs than in lateral spurs in all mutants, indicating their potential roles in spur morphological variation. The *SOB5* gene, involved in cytokinin biosynthesis, belongs to a family of plant-specific small proteins. Its overexpression elevates endogenous cytokinin levels by upregulating *AtIPT3/7*, significantly altering growth and hormonal homeostasis in *Arabidopsis* [34]. In *I. uliginosa*, *SOB5* expression was lower in the WT than in mutants, and significantly lower compared to 2SM, implying a possible function in variant spur formation. The *LTP*, *SOC1*, and *TCP4* genes each contribute to distinct aspects of plant development. *LTP* participates in wax or cutin deposition within expanding epidermal cells and secretory tissue cell walls. *SOC1*, a MADS-box protein closely tied to floral development, is widely studied in flowering and fruit maturation. *TCP4* has been established as a regulator of spur development in *Aquilegia* and *Linaria* [8, 9, 35, 36]. All three genes displayed a consistent expression pattern in *I. uliginosa*, with higher expression levels in the WT than in mutants, suggesting they may function similarly in the molecular regulation of spur variation. Furthermore, the TF *NAC2* showed elevated expression in mutants relative to the WT, with high levels in the main spurs of 2SM and 3SM. Given its known roles in leaf senescence, floral development, lateral root formation, and secondary wall thickening [37–39], *NAC2* may also be involved in regulating lateral spur formation in *I. uliginosa*. All genes involved in growth and development, floral development, cell elongation, division, cell cycle regulation, and hormone responses directly or indirectly influence cell division and elongation activities, and are likely to play pivotal roles in the variation of spur morphology in *I. uliginosa*. In future work, we will prioritize core candidate genes for subsequent in-depth functional experiments, including gene knockout and overexpression analyses, to further elucidate the underlying molecular mechanisms.

## Conclusions

This study investigated the developmental dynamics of floral spurs in WT and mutant *I. uliginosa*. Both spur types followed a typical sigmoidal growth curve, with lateral spurs significantly shorter than main spurs. Rapid spur development commenced at the early stage, decelerated at the middle stage, and stabilized at the anthesis stage. Cytological analyses revealed similar cellular development mechanisms in WT and mutant spurs: early spur formation depends mainly on cell division, whereas subsequent length increase relies predominantly on cell elongation; the formation of internal cell protrusions is largely driven by cellular anisotropy. In addition, transcriptome sequencing was performed on spurs of WT and mutant plants at the early stage. Cluster analysis and functional enrichment analysis of 8,592 differentially expressed genes were performed to screen candidate genes associated with spur variation. Our results indicate that hormones play a pivotal role in spur variation, whereas genes involved in cell elongation, division, and the cell cycle are among the most critical factors shaping spur morphology. This study offers the first molecular insights into spur variation in the genus *Impatiens*, providing valuable information and a theoretical foundation for understanding spur diversity in dicotyledonous plants.

## Methods

### Plant materials

Plants bearing a single floral spur were defined as WT of *I. uliginosa*, whereas those with two or three spurs were classified as mutants. Seeds of both WT and mutant *I. uliginosa* were collected from the area surrounding Kunming Laoyu River Wetland Park,located on the eastern shore of Dianchi Lake along East Huanhu Road, Kunming City, Yunnan Province. This site is a plateau lake-type wetland, with a central geographic location of approximately 24.83°N, 102.81°E and an elevation of about 1890 m. These seeds were cultivated in the greenhouse at Southwest Forestry University under controlled conditions of 18–25 ℃ with a photoperiod of 11–13 h of light per day.

### Observation of spur growth dynamics

Following the methodology described by Li et al. (2024), buds with uninitiated floral spurs were randomly selected from 30 healthy *I. uliginosa* plants and monitored daily, resulting in 30 biological replicates. The length of each spur was measured daily at 9:00 AM, with three technical replicates per measurement. Measurements were conducted consecutively for 15 to 25 days, depending on the developmental progression of the plants. Growth curves were plotted based on the observational data. Three key developmental stages were identified by analyzing the spur growth curves in both WT and mutant *I. uliginosa*.

### Scanning electron microscopy and histological examination

Following the methodology of Li et al. (2024), tissue samples were fixed in FAA (a mixture of 50% ethanol, glacial acetic acid, and 38% formaldehyde at an 18:1:1 ratio). After dehydration through an ethanol gradient, the samples were critical-point dried with carbon dioxide using an EMS 850 dryer (Hatfield, PA, USA) and then imaged with a Zeiss Sigma 300 scanning electron microscope (Oberkochen, Germany). Following another ethanol gradient dehydration, the tissues were cleared in xylene, embedded in paraffin, and sectioned. Sections of 8 μm thickness were stained with 0.01% Safranin O and 50% Fast Green before being imaged under a Leica DM750 optical microscope (Wetzlar, Germany).

### RNA sequencing and de novo assembly of transcriptome

Following the methodology of Li et al. (2022), nectary spurs from three developmental stages of both WT and mutant *I. uliginosa* were collected. Immediately upon excision, the spurs were preserved in liquid nitrogen. Each sample was mixed with more than three biological replicates. Total RNA was extracted using Plant RNA Purification Reagent for plant tissue (Invitrogen, Carlsbad, CA, USA), followed by genomic DNA removal with DNase I (Takara). Subsequently, RNA-seq libraries were prepared using the TruSeq™ RNA Sample Preparation Kit (Illumina, San Diego, CA, USA). After quantification with a TBS380 fluorometer using Picogreen, the libraries were sequenced in a single lane on an Illumina HiSeq X Ten or NovaSeq 6000 platform (Illumina, San Diego, CA, USA) to generate 2 × 150 bp paired-end reads.

The raw paired-end sequencing data were trimmed and quality controlled using SeqPrep (https://github.com/jstjohn/SeqPrep) and Sickle (https://github.com/najoshi/sickle) with default parameters. Clean data were de novo assembled using Trinity (http://trinityrnaseq.sourceforge.net/), and homologous clustering of the assembled transcripts was performed, with the longest transcript within each cluster defined as a unigene [40]. The assembled sequences were further filtered for optimization using TransRate (http://hibberdlab.com/transrate/), redundant and similar sequences were removed using CD-HIT (http://weizhongli-lab.org/cd-hit/), and the completeness of the transcriptome assembly was evaluated using the Benchmarking Universal Single-Copy Orthologs tool (BUSCO, http://busco.ezlab.org).

### Functional annotation

All assembled transcripts were functionally annotated by querying the NR, Swiss-Prot, Pfam, and COG databases. BLASTX was used to identify proteins with the highest similarity to the given transcript sequences, with a cutoff E-value typically set to less than 1.0 × 10⁻^5^. GO annotations for the unique assembled transcripts were obtained using the BLAST2GO program (http://www.blast2go.com/b2ghome) [41] to describe biological processes, molecular functions, and cellular components. Metabolic pathway analysis was conducted using the KEGG database (http://www.genome.jp/kegg/) [42].

### Differential expression analysis and functional enrichment

The expression levels of genes and transcripts were calculated using the TPM (transcripts per million reads)/FPKM (fragments per kilobase of transcript per million reads) method, and the quantification of gene and transcript abundance was performed using RSEM (http://deweylab.biostat.wisc.edu/rsem/) [43]. Differential expression analysis was conducted using DESeq2 [44] /DEGseq [45] /EdgeR [46], with a threshold of Q value ≤ 0.05, |log2FC|> 1, and genes with Q value ≤ 0.05 (DESeq2 or EdgeR)/Q value ≤ 0.001 (DEGseq) being considered significantly differentially expressed.

GO and KEGG functional enrichment analyses were performed on differentially expressed genes. GO functional enrichment analysis was carried out using Goatools (https://github.com/tanghaibao/GOatools), while KEGG pathway analysis was performed using KOBAS (http://kobas.cbi.pku.edu.cn/home.do) [47]. Fisher's exact test was utilized for calculations, and *P*-values were corrected using the Bonferroni and BH (FDR) methods. The threshold for adjusted *P*-values was set at 0.05.

### Identification of TFs and cluster analysis

Through HMMER analysis, the domain information of transcripts was compared with the PlantTFDB (http://planttfdb.cbi.pku.edu.cn/) database to obtain homologous TF information for gene TF prediction and family analysis. Hierarchical clustering was conducted on differentially expressed TFs.

### qRT-PCR analysis of gene expression

Total RNA was extracted from five spur types of both WT and mutant *I. uliginosa* using the E.Z.N.A.® Plant RNA Kit from Omega. The first-strand cDNA was synthesized as a template using the EasyScript® One-Step gDNA Removal SuperMix from TransGen. qRT-PCR amplification was performed using the Roche LightCycler®480 II real-time quantitative PCR detection system, in combination with the Hieff® qPCR SYBR® Green Premix from Yisense. Details of the qRT-PCR amplification primers are provided in Supplementary Data Table S7, with *IuActin* serving as the reference gene. The fluorescence quantitative PCR detection utilized a three-step method, with three technical replicates for each sample. The amplification protocol was as follows: pre-denaturation at 95 °C for 5 min, followed by 40 cycles (denaturation at 95 °C for 10 s, annealing at 60 °C for 20 s, and extension at 72 °C for 20 s). Gene expression levels were calculated using 2^−ΔΔCt^ method.

### Statistical analysis

All experiments were repeated with three biological replicates. Statistical comparisons were performed using one-way analysis of variance (ANOVA), and post-hoc multiple comparisons were carried out via Duncan’s multiple range test. All statistical analyses were completed using SPSS 26.0. The significance threshold was set at *P* < 0.05. In figures and tables, different lowercase letters indicate significant differences among treatments at *P* < 0.05, while the same lowercase letter represents no significant difference.

## Supplementary Information

Supplementary Material 1.

## Data Availability

The original transcriptome sequencing data have been submitted to the NCBI BioProject database with accession number: PRJNA1422964.

## Abbreviations

NR: NCBI Non-Redundant Protein Sequence Database
Pfam: Protein Families
COG: Clusters of Orthologous Groups of proteins
GO: Gene Ontology
KEGG: Kyoto Encyclopedia of Genes and Genomes
